# The spectrum of *in vitro* maturation in clinical practice: the current insight

**DOI:** 10.3389/fendo.2023.1192180

**Published:** 2023-06-26

**Authors:** Mohd Faizal Ahmad, Marjanu Hikmah Elias, Norazilah Mat Jin, Muhammad Azrai Abu, Saiful Effendi Syafruddin, Ani Amelia Zainuddin, Nao Suzuki, Abdul Kadir Abdul Karim

**Affiliations:** ^1^ Department of Obstetrics and Gynecology, Faculty of Medicine, National University of Malaysia, Kuala Lumpur, Malaysia; ^2^ Faculty of Medicine Health Sciences, Universiti Sains Islam Malaysia, Nilai, Malaysia; ^3^ Department of Obstetrics and Gynecology, Faculty of Medicine, Universiti Teknologi MARA, Jalan Hospital, Sungai Buloh Selangor, Malaysia; ^4^ Medical Molecular Biology Institute, National University of Malaysia, Kuala Lumpur, Malaysia; ^5^ Department of Obstetrics Gynecology, St Marianna School of Medicine, Kawasaki, Japan

**Keywords:** *In vitro* maturation (IVM), *In-vitro* fertilization (IVF), cryopreservation, oocytes, maturation

## Abstract

*In vitro* oocyte maturation (IVM) has been used worldwide. Despite the long-term implementation, the uptake of this procedure to complement current *in vitro* fertilization (IVF) remains low. The main reason is likely due to the non-synchronization of protocol and definition criteria, leading to difficulty in collective proper outcome data worldwide and, thus, lack of understanding of the exact IVM procedure. The review aims to consolidate the current clinical practice of IVM by dissecting relevant publications to be tailored for a current spectrum of clinical practice. Nevertheless, the background theories of oocyte maturation were also explored to provide a comprehensive understanding of the basis of IVM theories. Additional discussion of other potential uses of IVM in the future, such as in ovarian tissue cryopreservation known as OTO-IVM for fertility preservation and among women with diminished ovarian reserve, was also explored. Otherwise, future collaboration among all IVM centers is paramount for better collection of clinical data to provide valid recommendations for IVM in clinical practice, especially in molecular integrity and possible DNA alteration if present for IVM offspring outcome safety purposes.

## 1 Introduction

The oocyte maturation process consists of complex steps with a composed intrinsic capacity that aims to support normal fertilization (1). The optimum environment maintains the process *in vivo*, thereby leading to the extrusion of meiosis II (MII) stage oocytes at the end of the ovarian cycle (2). Nevertheless, this phenomenon might differ in infertile women leading to sub-optimal oocyte maturation and thus failure to fertilize (3). Therefore, implementing *in vitro* maturation (IVM) using special media and technique can overcome this issue in selected cases. However, the IVM of oocytes is a complex and dynamic process (4). Therefore, it is a difficult but reproducible technique following an established protocol with a variable outcome. The idea of IVM emerged more than 50 years ago and has been applied since then. The established methods of IVM are based on the co-incubation of cumulus–oocyte complex with activated granulosa cells (GCs). Although IVM had demonstrated the progression of the cell cycle achieving MII oocytes with significant breakthroughs in human reproduction in early 1990 following live birth, it is still considered an experimental technique due to the inconsistency of the results. The maturation, fertilization, and fertilization rates mainly depended on the type of IVM protocol. Although various protocols had been explored and implemented, the outcome was mostly similar—at least 30% to 40% (5, 6). In addition, the new strategy for ovarian tissue cryopreservation (OTC) was to add the IVM for the immature oocytes harvested during cortical dissection found to have better outcomes with at most 50% maturation rates (7). However, overall fertilization and blastocyst rates are similarly reported—30% to 40% (8, 9). Thus, IVM outcome is still considered as inconclusive (10). Moreover, most of the early eras of implementation of IVM in humans are different from an excellent clinical outcome, mainly poor development potential following insemination. Following this, the progression of the IVM technique has been reported in the human reproductive field, aiming to create an optimum medium mimicking the physiology environment *in vivo* (11, 12). Currently, most IVM centers use self-prepared media considering that commercialized media are limited due to evolving medium composition and still experimental in nature (13, 14). To date, these media had been evaluated via extensive research utilizing most antioxidants (e.g., glutathione and growth-promoting factors): follicular-stimulating hormone (FSH), luteinizing hormone (LH), and steroids, which aim to promote synchronization of nuclear and cytoplasmic maturation (15, 16). Conversely, the combination of the medium preparation remains a mystery; thus, expectedly, the oocyte quality, fertilization, and pregnancy rates following IVM are still limited worldwide (15). Given this reason, most IVM centers have intensively dissected its technique, thereby sharing molecular evidence to improve the current IVM outcome. To date, the introduction of new steps for oocyte activation before the IVM procedure has been established. The pre-IVM steps are known as pre-maturation or capacitation cultured medium with C-type natriuretic peptide (CNP) called CAPA culture (17, 18). This step utilized CNP to induce the stage-dependent maturation features in the oocytes retrieved from sub-optimal antral follicles, likely less than 10 mm (18, 19). Nevertheless, CAPA-IVM steps theoretically provide promising results as the culture system added pre-maturation culture combination components, such as CNP, estradiol (E2), and insulin (20, 21). Most importantly, it can be done in a non-stimulated ovary with better oocytes and embryo quality than standard IVM. Nevertheless, controversy regarding the spectrum of IVM clinically has been debated mostly for its proper definition and implementation (10, 22). For example, the type and steps of medium combination, stimulation regime, and its current progression technique are much broader nowadays (22). Therefore, our review aims to consolidate the current evidence-based IVM for clinical application for better understanding and insight, thus concluding as a recommendation that IVM could offer for a better future implementation.

## 2 Controversial definition of *in vitro* maturation

However, the standard definition should be clarified. Most of the centers had defined their IVM procedure based on a local protocol. Thus, the synchronization of definitions leads to the clarity in concluding the overall outcome in the future. The definition’s main concern was determining the safety of the IVM outcomes. However, as most centers had offered IVM, accurate data cannot be pulled because of the non-standard definitions, particularly in dissecting the gene expression or DNA imprinting and integrity following IVM (10, 22). Therefore, a proper standard definition should be highlighted to ensure that comparable IVM outcomes can be determined from multiple centers that aim for a proper evaluation of the safety of IVM outcomes in the long term and are reliable worldwide (23). Interestingly, translational research has been established to support the proper definition of IVM with scientific clarification and supporting evidence.

### 2.1 Standard IVM

Following the early implementation of IVM, it caters to immature oocytes collected from non-stimulated ovaries (24). However, gonadotrophin stimulation was primarily adopted from animal cases during the early 1970s (22, 25). Subsequently, the protocol of IVM coupled with the gonadotrophin ovarian stimulation cycle has been widely implemented because it also yields immature oocytes that require an IVM protocol (26). At that time, the immature oocytes—germinal vesicles (GV) and meiosis I (MI), were able to mature *in vitro*, thereby yielding MII oocytes that are usable for fertilization. Nevertheless, during these cycles, no human chorionic gonadotrophin (hCG) trigger was given because it is aimed to yield immature oocytes, although it was a stimulated ovarian stimulation. Therefore, any cycle utilizing IVM protocol with or without ovarian stimulation and not using any triggering agent should be defined as standard IVM (27). This definition is tailored to the early introduction of IVM in the human field and should remain the standard as it mimics the natural physiology of the ovary as LH surge is happening naturally for ovulation process rather than externally triggered via hCG injection.

### 2.2 Non-standard IVM

**Figure 1:**
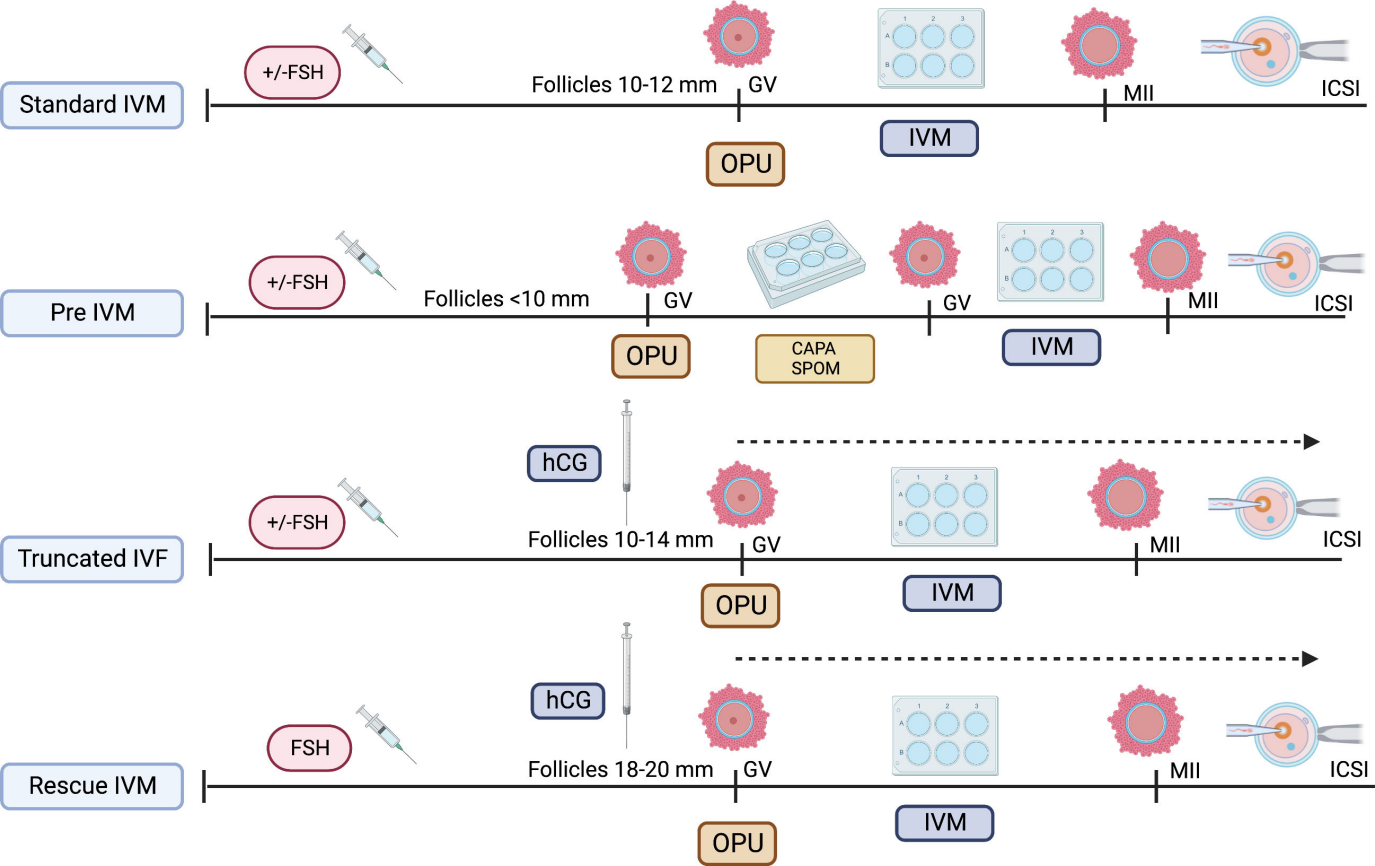
Current IVM protocols.

As the IVM protocol was adopted worldwide, the modification of its protocol emerged (27). The idea of oocyte nuclear maturation using trigger agent, hcg, had become widespread, thereby aiming to improve the overall IVM outcome clinically (28, 29). Although the initial understanding suggested that the LH receptor was not active in smaller size follicles, the outcome was contradictory. Most stimulated ovarian cycles with triggering agent result in cumulus expansion and GV breakdown (GVBD), thereby leading to multiple stages of oocyte maturation—GV, MI, and MII in one cycle (30). Therefore, it does not exclusively produce immature oocytes, although triggers were given during smaller follicles, primarily 10–12 mm. Over the years, most translational research has found that utilizing the hcg trigger at smaller follicles jeopardize the overall IVM outcome postulating that the potentially negative impact of these trigger on immature oocytes leads to poorer development of *in vitro* mature oocytes as compared to *in vivo* mature oocytes in same cycles (18, 21, 22). Therefore, although most centers adopt this protocol, labeled as IVF/M cycles, the outcome remains low to be translated into clinical pregnancy, although utilized among normal ovarian reserve women (31). Nevertheless, it can be reserved as an alternative fertility preservation (FP) strategy, primarily for women with a limited stimulation window, such as patients with cancer aiming for more mature oocytes for future use (32). Thus, utilizing IVM protocol in cycles that potentially harvest both immature and mature oocytes in the same cycle and using the hcg trigger are currently not considered standard IVM because not all oocytes were mature *in vitro* per se (10, 22). Furthermore, most evidence currently recommends the term “rescue IVM” or preferably “truncated IVF” as multiple numbers of insemination were done in the same ovarian stimulation cycle, depending on the oocyte maturation stage. In this cycle, all immature oocytes will be cultured with IVM media, whereas mature oocytes will be inseminated with sperm as in standard IVF cycles. Therefore, this is not considered physiology as initiation of oocyte maturation had been initiated with external hCG injection and thus should be defined as non-standard IVM (10, 22). Otherwise, the diversity of IVM protocols is illustrated in **Figure 1**.

## 3 Physiology of oocyte maturation

The importance of oocyte maturation is the synchronization of nuclear and cytoplasm maturation (33, 34). The prerequisite to prime this process should be catered to the ultrastructure and organelle of these immature oocytes to ensure an optimum response with the microenvironment of IVM culture, thereby resulting in a mature quality oocyte (35). Therefore, the fundamental physiology of oocyte maturation should be met by mimicking the *in vivo* process.

### 3.1 *In vivo* maturation

**Figure 2:**
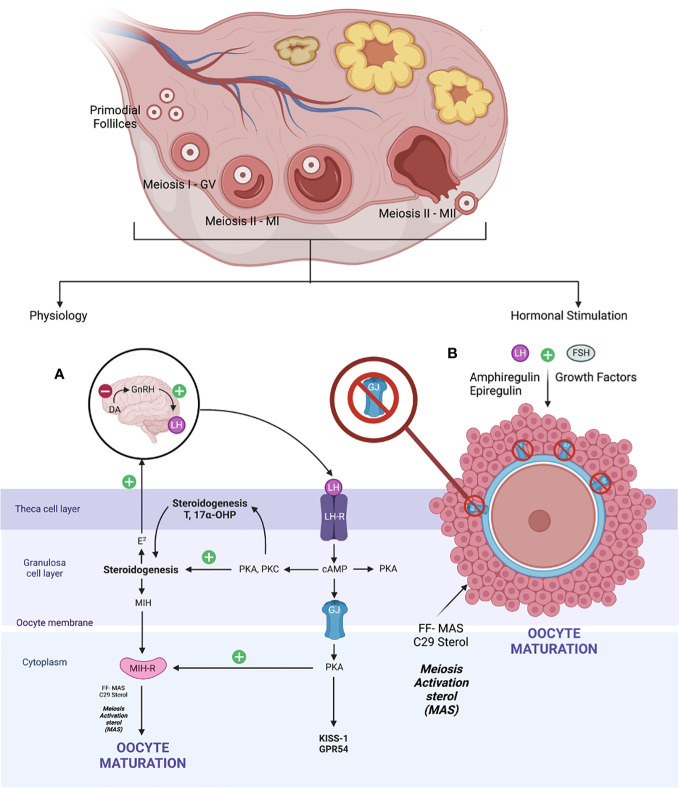
*In vivo* maturation physiology and hormonal stimulation.

The fundamental of oocyte maturation is uplifting the arrest of the PI stage to the MII stage via molecular factors that regulate the balancing of this condition. Most repressive mechanisms maintain by increasing intracellular cyclic adenosine (36) monophosphate levels that quiescent the maturation environment through the G protein–mediated stimulation of adenylyl cyclase and inhibition of intracellular phosphodiesterase (PDE) (37). Otherwise, gonadotrophin, growth factors, sterol, and steroid molecules trigger oocyte maturation, thereby downregulating these inhibitory precursors and resulting in mature oocytes. At the beginning of life, the primordial germ cells arise from the endoderm of the yolk sac, which migrates to the urogenital ridge via the hindgut. Production of oogonia begins with primary oocytes divided by mitosis, covered with flat epithelial cells (primordial follicles). Subsequently, the DNA duplication occurred during MI. At this point, the GV stage contains a large nucleus size. It was arrested in the diplotene stage of prophase I (PI) with subsequent resumption after ovulation—puberty stage leading to protrusion of MII oocytes—*in vivo* mature oocytes ready for fertilization (38, 39). When established, the exposure of LH that leads to the maturation of oocytes via steroidogenesis triggers the growth factors, leading to the formation of follicular fluid-derived meiosis, thereby activating sterol (FF-MAS)—C29 sterol that helps to disrupt the oocyte–GC complex supporting oocyte maturation and ovulation (40). Therefore, the role of C29 sterol supported oocyte maturation *in vitro* and stabilized MII oocytes before fertilization (41). The schematic of the *in vivo* maturation process of physiological and hormonal stimulation is defined in **Figure 2**. The novel IVM media and the protocol were developed by dissecting these microenvironments and adding the strategy aimed at enhancing the promoter of the oocyte’s maturation process.

### 3.2 Molecular aspect of oocyte maturation

However, the process of maturation is complex. In brief, it is initiated by the LH surge, leading to the cascade of chromatin condensation and breakdown of the GV (GVBD) stage. Following that, the MI starts and ends upon protrusion of the first polar body (PB1), harvesting one set of chromosomes with soon entering the MII cycle and arrest at metaphase II and will progress only if fertilization occurs—the mature MII oocytes (20, 33, 37, 39). These events are regulated by epigenetics and signaling pathways that maintain the maturation and quality of oocytes produced at the end of the cycle. Nevertheless, understanding the molecular aspect of the maturation of the oocyte is important. The initial molecules are essential in maintaining the state of mitotic arrest to ensure an excellent long fertility journey in women (42). The cyclic adenosine monophosphate (43) and natriuretic peptide precursor type C (NPPC) and receptor 2 (NPR2) are responsible for this matter. At the oocyte level, cAMP is regulated by adenyl cyclase, Gs protein, and G protein–coupled receptor 3. In contrast, in the cellular level—GC, cAMP is regulated by cyclic guanosine monophosphate (cGMP), which decreases the level of PDE3A activity that indirectly maintains the cAMP level in the oocyte environment. The cGMP is from GTP via guanylyl cyclases at both mural GCs (MGCs) and cumulus GCs (CGCs) (44). The coordination of cGMP from GC and oocytes environment via NPPC and NPR2 is vitally leading to sustainable cAMP for complete miosis arrest *in vivo*, primarily the spontaneous GVBD in oocyte–cumulus complex (COC). Alteration of this signal with a low level of cGMP can lead to a premature resumption of meiosis, thereby leading to fragmentation of oocytes with subsequently poor embryo development (45). Therefore, this is considered as important stage that triggers most of research interest to prevent this stage to happen prior to IVM process in clinical setting explained via CAPA-IVM process. However, it is still consider challenging to be mimic in IVM protocol due to complex mechanism involved not solely by additional CNP culture. Thus, the ongoing research to answer this research gap is still in progress.

**Figure 3:**
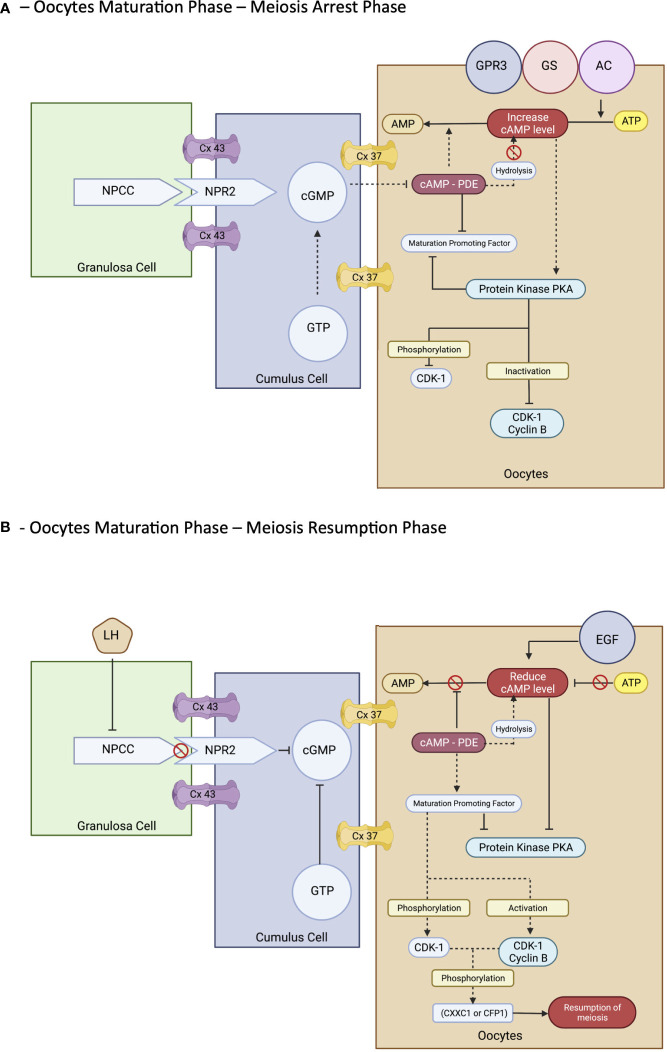
Meiosis phases in oocyte maturation.

In exploring the role of the NPPC/NPR2 system, most studies found that it was regulated by the FSH as well as the sex hormones receptor: primarily estrogen (ER) and androgen (AR) (38, 46–49). This theory explains that, in women with polycystic ovarian syndrome (PCOS), higher estrogen and androgen levels increase NPPC/NPR2 concentration, leading to mostly constantly low numbers of oocyte extrusion–anovulatory cycles (50). This phenomenon also reflects the poor outcome among women with PCOS clinically despite repeated IVF cycle; thus, IVM had been proposed as a good strategy for them. Nevertheless, the role of transforming growth factor–b also contributed to increasing NPPC levels; thus, current evidence recommends integrating it into the culture system to improve oocytes’ competency that recovered from small antral follicles (51). Conversely, a significant surge of LH is needed before the extrusion of oocytes. Profoundly, it leads to the downregulation of the NPPC/NPR2 system and decreased ER and AR levels, thereby inducing the maturation process. Furthermore, the concentration of PDE3A level will increase and continue to degrade the cAMP, leading to the upregulation of the epidermal growth factor (EGF) network in MGC/CGC. Subsequently, it activates the maturation-promoting factor (MPF) that promotes the resumption of meiosis via protein phosphorylation–anaphase-promoting complex and initiates the GVBD and, finally, the division of chromosomes (38, 44, 51). The role of the cyclin-dependent kinase 1 (CDK1) and cyclin B complex should be explored in dissecting the MPF activity. During the meiosis arrest stage, MPF was made quiescent by cAMP via protein kinase A regulation. Therefore, CDK1 facilitates chromosome condensation during the maturation stage via phosphorylation of CXXC- finger protein 1 responsible for deletion of SET domain containing 1–CCXC1 complex from chromatin during the resumption of meiosis (51). Thus, the CDK1 activation is paramount in initiating oocytes meiosis that was inhibited by a higher level of cAMP (20, 51). Regarding cytoplasmic maturation, it is essential for oocyte quality primarily via mRNA activation and regulation with proper organelles arrangement following meiosis resumption. During this time, there is an ultimate consumption of mRNA for protein synthesis, spindle assembly, and maintenance of the MII arrest as illustrated (**Figure 3**). The meiosis arrest female 1 is one of the crucial regulators reported for oogenesis, aiming for healthy offspring because it suppresses specific transcripts based on the desired level (52). The organelles’ arrangement comprises the maturation of cortical granules, mitochondria, endoplasmic reticulum (ERs), and the cytoskeleton. In brief, the cortical granules contained will be released at the perivitelline space leading to modification of the zona pellucida environment, thereby preventing polyspermy upon fertilization (53). Whereas the mitochondria will be the power source of energy via ATP supply, ER is responsible for Ca2+ regulation during fertilization (54). The meiosis phases in oocyte maturation are summarized in **Figure 3**. Impairment of these mechanisms will lead to poor oocyte quality. Nevertheless, poor or absence of fertilization was found because of suboptimal cytoskeleton rearrangement, leading to failure of chromosome division and transportation; thus, oocytes are arrested at MII following fertilization. Hence, inconsistency of IVM outcome is postulated to be part of this process, and a targeted outcome for a good oocytes’ quality following IVM is still challenging in the clinical setting.

### 3.3 Optimum environment for IVM

Evidence of molecular research has improved the current IVM research cohort technique, primarily in altering the media preparation. Although the theories evolved 10 years ago to consolidate the optimum environment *in vitro*, mimicking the *in vivo* maturation, the evolution persists as most centers opted for different protocols depending on subject matters: animal or human model (2, 10, 24, 28, 29). As explored, the essential physiological oocyte maturation should meet the balance of bioenergetics and metabolics for better cytoplasmic maturation, thereby maintaining a good interaction in the cumulus–oocyte complex in preserving the microenvironment factors. This aims to enhance oocytes’ maturation competency, thus stimulating the activation of somatic cells’ gene expression and metabolic changes, thereby leading to the successful initiation of the ovulation process (19, 38, 51). These three crucial vital processes should be imitated in IVM oocytes to improve their overall outcomes. The most critical hurdle in IVM oocyte development is maintaining meiosis arrest following oocyte extraction. However, a sudden decrease in cAMP will occur because of metabolic alteration, leading to the premature resumption of meiosis. If this occurs, then it will promote a desynchronized nuclear to cytoplasmic maturation, thereby either leading to poor oocyte quality or failure of maturation *in vitro* (18, 21, 34). One of the postulations of these conditions points toward the degree of FF-MAS exposure in the follicular fluid before retrieval. The volume of FF-MAS might be low as the follicle is still immature. However, the vast maturation process is the trigger in the follicle stage because of the activation of transcripts for protein synthesis, followed by the post-translational mechanism and genome activation, which primarily required a certain degree of FF-MAS exposure (40, 55). Thus, failure to achieve this will lead to spontaneous premature maturation in the cytoplasm while delaying nucleus maturation. Therefore, most literature do recommend that the IVM culture with follicular fluid improves the overall oocyte outcome although some in the animal model (56, 57). In addition, the COC should be retained with minimal denudation in maintaining the gap junction (44), and connexons interaction to ensure the cAMP level is maintained throughout the process, complementary with specific IVM culture media (21, 23). These culture media should contain natriuretic peptides (NPPC) or cAMP hydrolysis inhibitors with or without natriuretic peptide guanylate cyclase B–NPR2 to maintain cAMP level, thereby ensuring the state of meiotic arrest, thus preventing the premature resumption of meiosis. Direct cAMP analogs such as dibutyrylcyclicAMP (dbcAMP) improved oocyte maturation in animal models because this modulator can improve cAMP levels similarly to *in vivo* environments (15). Moreover, using cAMP inhibitors such as PDE inhibitors can reduce cAMP hydrolyses such as isobutylmethylxanthine (IBMX) and milrinone or cilostamide that act as specific PDE3A inhibitors that also help in maintaining the cAMP level *in vitro* (2, 10, 13). Subsequently, after incubation with this factor, the current composition of IVM media is primarily protein. Gonadotropin hormone–FSH can be added by lowering the PDE3A inhibitor or cAMP inhibitor level. These allow a slow maturation process mimicking the natural environment, thereby improving the overall oocyte quality, fertilization ability, and blastocyst formation. However, although it is currently promising, the ideal environment remains inconclusive as evidence is yet to be consolidated. Therefore, using a two-step strategy *in vitro* culture environment is considered optimum (17, 18).

## 4 Current implementation of *in vitro* maturation practice

**Figure 4:**
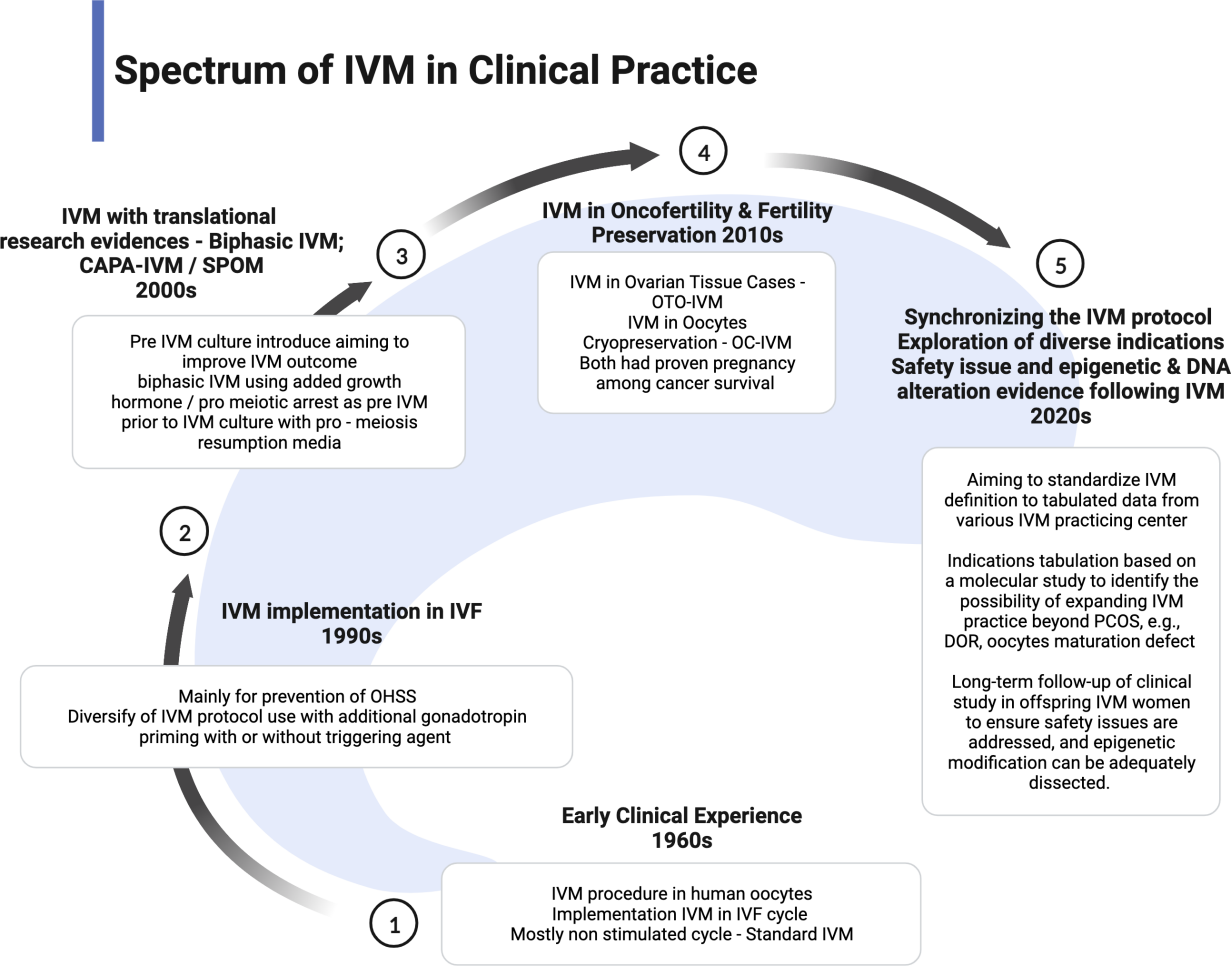
Spectrum of IVM in clinical practice.

The IVM practice is low uptake in most fertility centers because of low efficacy and pregnancy outcome. However, IVM has become one of the proper techniques for patients with cancer as the spark of FP services. Therefore, most centers with research facilities implemented the IVM project to enhance the efficacy and explored the epigenetic and fundamental techniques to improve the outcome (10, 58). More evidence is emerging in improvising the IVM protocol. Proper IVM definition and clinical practice implementation has been made to ensure synchronized data can be collected for future reference and comparison (22). However, most international societies still consider IVM as research-based at this point. Therefore, the IVM protocol is still being modified based on current findings or emerging from animal models, aiming to improve the overall outcome. The utilization of translational research such as CAPA-IVM is a current breakthrough in the IVM field because it offers reasonable modification of IVM preparation in two stages and proves to increase the maturation yield of oocytes and live birth (17, 18). As a pilot study, although the sample size is small, the theory is clinically proven. The indication of IVM has become more diversified. Therefore, the clinical implementation should be widened and modified based on the indication and possible modification. The current spectrum of clinical practice of IVM is summarized in **Figure 4**.

### 4.1 PCOS

For women with PCOS, it is considered that the ultimate strategy is to significantly reduce the risk of ovarian hyperstimulation syndrome and possible ovarian torsion caused by an enlarged ovary during stimulation. For over a decade, IVM has been used for the PCOS cohort as low-cost ART, either with minimal stimulation or natural cycles (59–62). However, the efficacy of IVM-PCOS remains low. The main reason was that the IVM protocol was not standardized worldwide for PCOS, as most centers using the non-standard IVM with prolonged priming and triggering agent. Therefore, the introduction of CAPA-IVM among PCOS cohorts in standard IVM protocol shows good efficacy, particularly in lower follicles <6 mm (17, 18). In addition, it improves maturation, as well as blastulation rates and live birth rates. This remarks that the standard IVM is the best option for this cohort. Moreover, specific phenotypes of PCOS also influence the overall IVM outcome. As reported, phenotype A PCOS had a better cumulative live birth with IVM than other phenotypes classified based on National Institutes of Health (NIH) consensus panel criteria (63). Moreover, systematic evidence does conclude that higher birth rates was seen among IVM protocol in women with PCOS with the clinical pregnancy and that implantation rates were higher, whereas cancellation rates lower maturation and miscarriage rates did not differ with non-PCOS women who underwent IVM cycles (64). Therefore, combining these factors can help improve IVM outcomes in the PCOS cohort in the future, based on current findings.

### 4.2 Women with cancer: fertility preservation

The option of oocyte cryopreservation (OC) among women with cancer before possible gonadotoxic primary cancer treatment had been implemented worldwide (65). The OC can be done with or without FSH priming, depending on the FP time frame given by the oncologist. Therefore, the number of mature oocytes is the key to good OC outcome. Goldman et al. proposed that the number of MII oocytes predicts the likelihood of live birth depending on the age (66). Therefore, a greater number of mature oocytes are the aim. However, the number of mature oocyte yields is low in a limited stimulation period. Thus, IVM can be an excellent strategy to enhance the number of mature oocytes. Although this IVM is considered truncated IVF, particularly, cancer cohorts, standard IVM has also been opted for. Nevertheless, the oocyte maturation rates (OMRs) following IVM in women with cancer were as high as 50% (67, 68). One of the postulations, most of these patients are not infertile patients; thus, oocyte maturation potential is optimum as compared to the subfertility cohort. Furthermore, the pregnancy rates following IVM in women with cancer were published (69–71). Therefore, IVM does improve the numbers of OC with proven pregnancy outcomes; thus, it can be offered as a good strategy for FP. In addition, emerging evidence has proposed the combination of OTC with OC (72). In the latest systematic review, Ahmad et al. recommended the combination of OTC with OC by utilizing the IVM (7). Most of the adolescent cancer cohort had achieved puberty; however, because of a short time, especially in hematological cancer, OTC is the preferred FP. During the procedure, most of their ovarian cortex contains oocytes during cortical dissection. As the OTC is generally done in the unstimulated cycle, most authors reported immature oocyte isolation during the OTC procedure. Hence, IVM culture was used, and a promising result had established (73). Most authors had at least 30%–40% OMR following IVM in oocytes harvested via the OTC (7). Therefore, FP outcomes are better with these combinations. Moreover, pregnancy had been reported using IVM oocytes harvested during OTC in women with breast cancer; thus, it concurs with the current recommendation (7, 73).

### 4.3 Diminished ovarian reserve

The dilemma of managing diminished ovarian reserve persists. Most centers offered minimal stimulation to yield more good quality oocytes, thereby increasing the success of clinical pregnancy and live birth (74). The ability to conceive among women with diminished ovarian reserve (DOR) via IVF is similar to regular, provided that a good quality of oocytes is obtained. It yields an acceptable embryo quality during embryo transfer. Nevertheless, various strategies have been implemented to improve DOR outcomes, primarily the anti-oxide supplement and adjunct therapy. None of these measures shift the landmark of practice in women with DOR (75). Moreover, poor quality of oocytes with the diversification of maturation oocytes stages is the ultimate problem among women with DOR who underwent standard control ovarian hyperstimulation (76). At least 15%–30% of oocytes yield was immature: GV and MI stages (77). Therefore, not utilizing these oocytes despite minimal stimulation leads to devastation and recurrent cycle failure. Therefore, the number of oocytes influences the success of IVF cycles as compared to women with usual ovarian reserve (NOR). This scenario sparks interest in IVM strategy for women with DOR (76, 78). Emerging evidence has consolidated the outcome of IVM among women with DOR. Unexpectedly, some authors reported that the OMRs were higher for DOR, particularly in GV-stage oocytes (76). Although most are observational studies with smaller samples, it can be an excellent way to improvise the strategy for women with DOR. Nevertheless, IVM among DOR primarily rescues IVM with an optimum dose of FSH priming and a triggering agent, with most of the follicles achieving a mature size of 18–20 mm (77). Molecularly, utilizing a trigger agent with more prominent size follicles will lead to the premature resumption of meiosis, thereby leading to poor oocyte maturation in the IVM cycle (18). However, this was not seen in IVM women with DOR in most published articles (79, 80). The morphokinetics and dynamic of the DOR oocytes environment were noted to be different, with solid molecular evidence of premature luteinization. The postulation also reflects that the microenvironment at GC and oocytes level of women with DOR, primarily cAMP and its regulation factor, including MPF, might differ from NOR (51, 76). Few authors do favor longer culture time for DOR oocytes to improve maturation outcomes (76, 80). However, this needs further dissecting because the evidence is scanty and needs further epigenetic exploration and molecular conclusion. Lee et al. observed a better maturation of GV to MII in DOR compared to that in NOR within the DOR cohort using rescue IVM (76). The competency of these oocytes was also similar to NOR, thereby increasing the successful IVF/IVM cycle of women with DOR. The fertilization rates and embryo development are similar to NOR women. Therefore, the report provides excellent clinical insight into exploring more about IVM among women with DOR. Others also reported the same outcome utilizing rescue IVM for DOR (80). Theoretically, this postulated that the pre-IVM culture might have a different outcome for DOR than that for NOR (76, 80). Therefore, marked clinical effects were seen among DOR cultured with these regimes. Furthermore, consolidating these findings, they reported an excellent pregnancy following rescue IVM among DOR. Therefore, women with DOR have a new indication for the IVM technique; thus, it can be implemented as part of clinical practice once more shreds of evidence can be gathered.

### 4.4 Resistant ovarian syndrome

This unique syndrome considers one of the rare infertility causes often referred to as ovarian insensitivity syndrome or “*Savage syndrome*” (81). Molecularly, it describes as a failure of follicles development due to multilevel signaling failure of FSH or beta subunit receptors (82). Thus, no response toward GnRH stimulation leads to multiple abandoned cycles. Most evidence concluded that the CNP is essential as a follicle growth regulator (83). It regulated the cGMP and cyclic phosphate (84) levels synergistically with natriuretic peptide receptor–B to enhance follicle development and, subsequently, maturation (85). However, this signaling process was absent in resistant ovarian syndrome (ROS) women, leading to failure of follicle maturation. In addition, the mutation of the FSH receptor gene also contributes to this matter (82). Recently, the IVM technique has been offered to ROS women (22). Emerging evidence had reported a positive IVF-IVM outcome among them with a live birth rate at 15% per started cycle overall with 30% per patient (86). Therefore, IVM is considered a promising strategy approach for ROS women (87).

## 5 Current protocol of *in vitro* maturation

### 5.1 Stimulated versus non-stimulated cycles

To date, most IVM cycles started with ovarian stimulation. Although most literature favors non-stimulate cycle, natural as a standard IVM cycle, the role of oral induction agent or gonadotropin priming should be explored (10, 22). At this point, most stimulation aims for better follicular development, including meiotic initiation and competency of immature oocytes *in vivo*. The insight into stimulation cycles primarily arises from an animal model (25). Therefore, to date, the idea of FSH priming is not conclusive. Most authors agreed to minimal stimulation for at least 2–3 days with at least 75–150 IU daily starting on day 2 or 3 after menses or withdrawal bleeding (10, 14, 23, 26). However, it should be tailored to the IVM cohort as non-PCOS women had no better outcome with the stimulated cycle (21).

### 5.2 Does the size of follicles matter?

The size of follicles depends on the nature of the cycle. In the non-stimulated cycle, most centers prefer to harvest follicles less than 10 mm, whereas, in the stimulated cycle, 10–12 mm is generally the aim. However, the size of follicles also depends on the type of IVM culture used in the local setting. The latest evidence from an established IVM center primarily aims for lesser than 10 mm, thereby aiming for better miotic arrest upon retrieval (4, 17, 21). Generally, these centers used pre-IVM preparation to enhance the oocyte maturation response before the standard IVM culture. Recent evidence supports that the OMRs, embryo quality, and pregnancy rate were way better in smaller follicles lesser than 10 mm (21). However, maintaining the rate for these sizes in particular might be technically difficult using a single-lumen needle. The premature *in vivo* maturation also signifies the size of follicles. In the center that aims for larger follicle sizes, mostly 14–16 mm, the premature LH surge can occur, thereby leading to the premature resumption of meiosis *in vivo* before the oocyte retrieval, leading to the poor outcome of the standard IVM cycle. The level of cAMP will be decreased upon retrieval as well as Gj and Cx disruption, leading to increase hydrolysis of cAMP with desynchronization of nuclear-cytoplasmic maturation (44, 51). Thus, expectedly, there will be a poor OMR or quality following IVM. Therefore, most authors recommend 10–12 mm as the size of a good follicle for IVM tailored to the nature of the cycle and type of IVM media used in the center (88, 89).

### 5.3 hCG/Lh trigger versus non-hCG/Lh trigger

The use of triggering agents hCG and gonadotropin agonists are often used in IVM cycles worldwide—most available evidence favor using hCG as a triggering agent before oocyte retrieval (28–30). Combining the hCG trigger with a short course of FSH priming has become one of the commonest regimes utilized in most IVM centers. Furthermore, it is reported to increase pregnancy rates (29). Nevertheless, the priming of hCG is considered a non-standard IVM because it will lead to LH surge and *in vivo* maturation; thus, truncated IVF is a preferable term (10, 22). The outcome of using the triggering agent in IVM cycles has become inconclusive. Nevertheless, most evidence did not show any improvement in IVM outcome regardless type of trigger (18, 22). Therefore, much research has been done to dissect this matter, including the epigenetic mechanism of oocyte maturation. To date, most of the literature has supported that using hCG adversely affected the IVM cycle (18, 22). The idea of IVM culture will be jeopardized as molecularly hCG level will reduce NPPC/NPR2, which directly reduces the concentration of cAMP. In addition, hCG will indirectly increase PDE3A concentration, thus further decreasing the level of cAMP via hydrolysis (18, 21). These will lead to the extrusion of various maturation stages of oocytes, thereby reflecting premature meiosis resumption and poor oocyte maturation outcomes following IVM culture. Therefore, the meiosis arrest stage should be maintained to improve the IVM outcome. The current recommendation of using a co-factor to improve meiosis arrests, such as NPR2 and dbcAMP, as an optimal IVM environment will share some light that the triggering agent should not be used in standard IVM (17, 18, 52).

### 5.4 Pre-IVM culture strategy

The idea of pre–IVM culture is not new. It has been established primarily in animal studies and utilized in veterinary practice. The simulated physiological oocyte maturation (SPOM) was proposed a decade ago with an animal model (90). This protocol imitates the pre-IVM culture in the *in vivo* physiological environment using a cAMP modulator, such as IBMX. Subsequently, the extended IVM culture with a low dose of PDE3 inhibitor, such as cilostamide to enhance maturation. Throughout, the SPOM protocol improved the overall IVM outcome (90, 91). However, although the IVM technique has been a shift for humans for more than three decades, the efficacy of IVM remains low, particularly in translating into clinical pregnancy and live birth. Therefore, the pre-IVM culture has become one of the main interests in modifying the current IVM protocol in humans. The IVM center has now ventured for a better outcome by widening the human IVM protocol strategy utilizing this epigenetic evidence coupled with the current clinical practice as translational research. Following the SPOM, the capacitation–IVM (CAPA-IVM) has been proposed and shown a promising result in addition to a current standard IVM protocol (17, 18, 90). In CAPA-IVM, the homogenous oocyte cohort had been retrieved following a short course of FSH priming without a triggering agent. Most of the follicles were small to mid-antral (2–10 mm). Therefore, their process required a pre-IVM as a capacitation stage by incubating the immature GV stage oocytes with pro-meiosis arrest factor—CNP. This step aims to maintain the meiosis arrest stage by inhibiting premature resumption of meiosis. The later process will resume slowly, thereby aiming to improve the synchronization of nuclear and cytoplasmic maturation (18, 35, 40, 41). Subsequently, the culture continues with concurrent IVM medium with the presence of human recombinant amphiregulin, the EGF, which promotes oocytes meiosis resumption by reducing the concentration of cAMP by inhibiting CNP expression at GCs and NPR2 in cumulus cell (58). Overall, the CAPA-IVM offers promising results for point of interest, primarily fertilization, good oocyte quality, clinical pregnancy, and live birth. It also shows explicitly a significantly better result for small follicles of less than 6 mm (17, 18, 21). Thus, it can be a good strategy for women who do not require stimulation, such as women with PCOS or cancer contraindicated with stimulation or limited time intervals for stimulation before cancer treatment.

## 6 Future of *in vitro* maturation

IVM had engaged as part of ART based on progression, thereby aiming to improve the overall fertility outcome. Indications for IVM had emerged from PCOS to FP either in oocytes alone or combined with OTC (7, 10, 80). To date, women with DOR benefit from IVM in their ART cycle to increase the chances of mature oocytes for better pregnancy outcomes. The concept of “golden eggs” should be highlighted for DOR. The fertility outcome depends on the number of mature oocytes obtained in their IVF cycle; thus, it can be a good cohort of IVM users in the future. Although current evidence supports the use of IVM in DOR, the molecular aspect of maturation should be explored more because it might differ from that in NOR women; thus, the strategy can be unique for this cohort. The future establishment of a baseline of epigenetic characteristics with growth potential mechanisms can be a good reference in modifying the current IVM protocol for women with DOR per se. The evolution of pre-IVM culture also provides a good strategy to improve the non-stimulated–”standard IVM” outcome (10, 22). Nevertheless, without the pre-IVM culture, the “non-standard IVM” also helps in improving overall fertility outcomes, although it is not considered part of the mainstream IVM. Most authors support that the non-standard IVM primarily rescues IVM among poor responders or women with DOR. Therefore, the pre-IVM culture should be extended into various indications rather than women with PCOS alone to determine its efficacy in the future. In addition, the pre-IVM medium composition might differ in a different cohort of women, such as DOR, pure oocyte maturation defect—GV syndrome and OTO-IVM cohort (10). Future research can provide a good insight into modifying the pre-IVM media specific to the cohort. Thus, standard IVM with no stimulation and triggering can be implemented to synchronize the protocol to improve overall efficacy worldwide. Moreover, the IVM culture medium differs from all centers practicing it (12, 15, 16). Formula and recipes also differ based on continent and research team. Expectedly, the outcome is much different, and the overall result did not support a similar conclusion, leading to diverse evidence that remains inconclusive. Looking forward, the commercialized IVM medium that is standardized or similar medium preparation should be recommended to ensure synonymous, yet comparable outcomes can be offered similar to the current conventional IVF. The strategy can help consolidate the clinical implementation of IVM as a standard practice rather than remain experimental in the future.

Furthermore, the IVM should adopt a clear definition for future references, particularly in correlating it with epigenetic mechanisms or molecular evidence in consolidating the theories and outcomes (22). Regardless of whether “standard” or “non-standard” IVM is used, the definition should be highlighted. This is very important because it needs to dissect the safety issue of IVM, particularly in terms of DNA mutation or alteration following different culture procedures that can impair the offspring’s future. For example, the IVM with possible alteration of oocytes’ microenvironment might possess genetic imprinting abnormality that might not be apparent currently. Fortunately, available evidence finds no issue regarding neonates born via concurrent IVM procedure as compared to IVF alone. Nevertheless, this evidence is scatted and small; thus, more significant trials or discoveries with long-term follow-up should be encouraged. In addition, molecular evidence should be proven to ensure that IVM can be implemented safely rather than in clinical trials alone. Otherwise, a multicenter trial under the ultimate IVM consortium should be established. However, working in isolation will not concur the subject better. Opening more opportunities for collaboration in the center that is interested in participating is a way to go in the future. Thus, expanding the networking with the oncofertility center—mostly a research institute in a university setting can help gather more teams to work on IVM progression and establish a good team. OTO-IVM and OC-IVM are one of the primary services provided by the oncofertility consortium/team; thus, these collaborations can step up the progression of IVM research and implementation. For example, in Asia, although Vietnam is considered the leading country for IVM, emerging oncofertility in Asia such as Japan, India, Korea, and Malaysia is also interested in venturing into the IVM consortium for better IVM outcomes. Collaboration is the key to future success.

## 7 Conclusion

In a nutshell, IVM has come a long way with the modification of protocol adopted worldwide. Although the overall IVM outcome remains lower than conventional IVF, significant progress has been made in recent years, thereby leading to better implementation and live birth rates (10, 58). However, there is no doubt that the vast potential of IVM is yet to be translated into the human field. Therefore, further collaboration in multicenter with the establishment of the specific consortium is paramount to answering the significant research gap to identify the correlation of epigenetic and DNA modification following IVM that can lead to better oocyte maturation process in various cohort diseases. Furthermore, the latter can help widen the utilization of IVM without jeopardizing its outcome.

## Permission to reuse and copyright

This agreement - XE255AIVH2 is to confirm that Ahmad Mohd Faizal has been granted a license to use the BioRender content, including icons, templates, and other original artwork, appearing in the attached completed graphic pursuant to BioRender’s Academic License Terms. This license permits BioRender content to be sublicensed for use in journal publications.

## Author contributions

Conceptualization: AF, AK, ME, and NS. Data curation: AF, NJ, MA, AK, and SS. Formal analysis: AF, NJ, MA, AK, and AZ. Methodology: AF, ME, SS, and NS. Project administration: AF, AK, ME, and MA. Supervision: AZ, AK, and NS. Writing—original draft: AF, ME, SS, and MA. Writing—review and editing: AF, AK, ME, and AZ. All authors contributed to the article and approved the submitted version.
